# Supplementary material for: Assessment of Physical Tests in 6–11 Years Old Children: Findings from the Play Lifestyle and Activity in Youth (PLAY) Study
Source: Int J Environ Res Public Health. 2023 Jan 31;20(3):2552. doi: 10.3390/ijerph20032552 (PMC9915144; doi:10.3390/ijerph20032552)
Supplement: Supplementary file 1 [file ijerph-20-02552-s001.zip › ijerph-2159289-supplementary/Supplement Table S1.pdf]

Supplement Table S1. Obstacle course points system

	Points	Note	Possible Maximum points
**Single leg hop:**			
Landing in inner circle*	2 points	6 circles	12 points
Using the same foot in consecutive hops in each station	1 point	2 single hop stations	2 points
Completing the consecutive hops in each station without extra hop	1 point	2 single hop stations	2 points
**Double leg hop:**			
Demonstrating sound landing mechanics with good balance	2 points	3 hop-landings	6 points
Completing the consecutive hop- landing on both feet at the same time	1 point	1 double hop station	1 point
Performing consecutive hops without contacting objects¶	1 point	1 double hop station	1 point
**Kicking a ball:**			
Approaching a ball with continuous, steady running speed without stopping	1 point	1 kicking station	1 point
Final step: Taking a slightly longer step before previous step to prepare for kicking movement	1 point	1 kicking station	1 point
Kicking leg: Kicking a ball with smooth back-swing, downwards kicking motion, foot contact with a ball, and follow-through after kicking a ball	1 point	1 kicking station	1 point
Supporting leg: Planting firmly to assist entire kicking a ball motion	1 point	1 kicking station	1 point
A ball goes through center of two cones‡	3 points	1 kicking station	3 points
**Catching a ball:**			
Being able to stop at catching line with preparing to catch a ball	1 point	1 catching station	1 point

A thrown ball did not pass through hand(s) and hit other body parts	1 point	1 catching station	1 point
Catching a ball with either one hand or two hands	1 point	1 catching station	1 point
Catching a ball "cleanly" without fumbling or juggling	1 point	1 catching station	1 point
**Throwing a ball:**			
Showing an overhand throw with contralateral arm and foot movements	1 point	1 throwing station	1 point
Demonstrating a trunk rotation and a follow-through in the throwing motion	1 point	1 throwing station	1 point
Being able to complete entire throwing motion with a well-controlled manner	1 point	1 throwing station	1 point
A ball hits center of the target (screen)[§]	2 points	1 throwing station	2 points
**Sliding:**			
Being able to pick up a ball from a cone cleanly	1 point	6 cones	6 points
Being able to place a ball to a cone smoothly	1 point	6 cones	6 points
Maintaining sideway sliding motions to move to a right direction	1 point	1 throwing station	1 point
Maintaining sideway sliding motions to move to a left direction	1 point	1 throwing station	1 point
Being able to transfer a ball from picking up hand to placing hand	1 point	1 throwing station	1 point
Maintain knee bent, feet apart movements during sliding	1 point	1 throwing station	1 point
Performing sliding motions with low center of gravity	1 point	1 throwing station	1 point

*When foot was landed between inner and outer circles, 1 point was awarded.

[¶] The height of the object is 15.3 cm.

[‡] When a ball was passed between the middle two cones and outer two cones in each side, 2 points were awarded.

[§] When a ball did not hit center of the target, but somewhere between screen, 1 point was awarded.
