# Supplementary figures and images for: Assessment of Physical Tests in 6–11 Years Old Children: Findings from the Play Lifestyle and Activity in Youth (PLAY) Study
Source: Int J Environ Res Public Health. 2023 Jan 31;20(3):2552. doi: 10.3390/ijerph20032552 (PMC9915144; doi:10.3390/ijerph20032552)

Supplement Figure S1. Obstacle course diagram

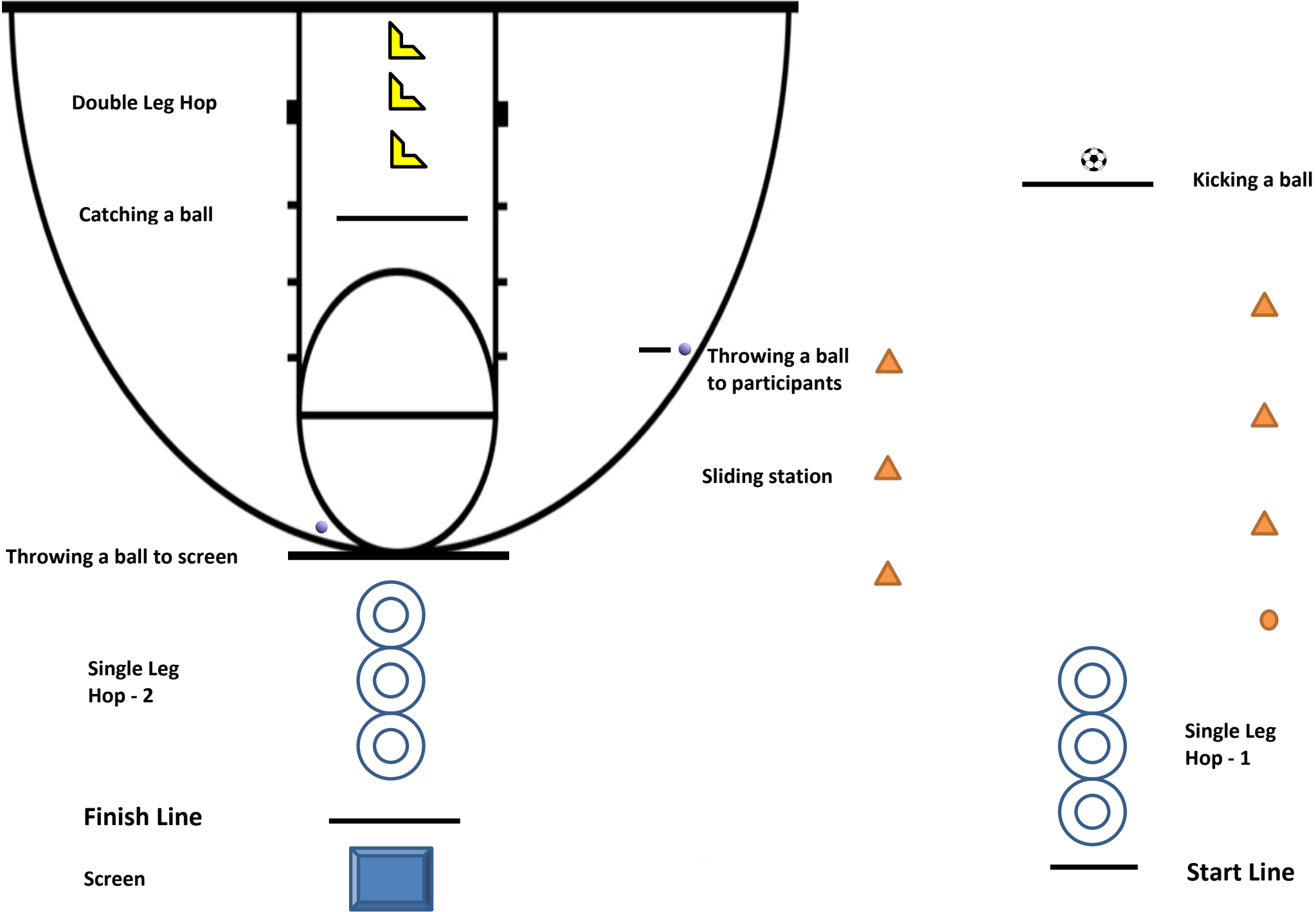

Supplement: Supplementary file 1 [file ijerph-20-02552-s001.zip › ijerph-2159289-supplementary/Supplement Figure S1.pdf]
